# Impact of Climate Change and Air Pollution on Bronchiolitis: A Narrative Review Bridging Environmental and Clinical Insights

**DOI:** 10.3390/pathogens14070690

**Published:** 2025-07-14

**Authors:** Cecilia Nobili, Matteo Riccò, Giulia Piglia, Paolo Manzoni

**Affiliations:** 1 SCDU Pediatria e Neonatologia, Ospedale Degli Infermi, Dipartimento Materno-Infantile ASLBI, 13875 Ponderano, BI, Italy; 2 Postgraduate School of Pediatrics, University of Turin, 10126 Turin, TO, Italy; 3 AUSL–IRCCS di Reggio Emilia, Servizio di Prevenzione e Sicurezza Negli Ambienti di Lavoro (SPSAL), 42123 Reggio Emilia, RE, Italy; 4 School of Medicine, University of Turin, 10124 Turin, TO, Italy

**Keywords:** climate change, air pollution, bronchiolitis, RSV, environmental health

## Abstract

Climate change and air pollution are reshaping viral circulation patterns and increasing host vulnerability, amplifying the burden of respiratory illness in early childhood. This narrative review synthesizes current evidence on how environmental exposures, particularly to nitrogen dioxide, ozone, and fine particulate matter, contribute to the incidence and severity of bronchiolitis, with a focus on biological mechanisms, epidemiological trends, and public health implications. Bronchiolitis remains one of the leading causes of hospitalization in infancy, with Respiratory Syncytial Virus (RSV) being responsible for the majority of severe cases. Airborne pollutants penetrate deep into the airways, triggering inflammation, compromising mucosal defenses, and impairing immune function, especially in infants with pre-existing vulnerabilities. These interactions can intensify the clinical course of viral infections and contribute to more severe disease presentations. Children in urban areas exposed to high levels of traffic-related emissions are disproportionately affected, underscoring the need for integrated public health interventions. These include stricter emission controls, urban design strategies to reduce exposure, and real-time health alerts during pollution peaks. Prevention strategies should also address indoor air quality and promote risk awareness among families and caregivers. Further research is needed to standardize exposure assessments, clarify dose–response relationships, and deepen our understanding of how pollution interacts with viral immunity. Bronchiolitis emerges as a sentinel condition at the crossroads of climate, environment, and pediatric health, highlighting the urgent need for collaboration across clinical medicine, epidemiology, and environmental science.

## 1. Introduction

Respiratory diseases represent a leading cause of pediatric morbidity, particularly within the first year of life. Acute lower-respiratory-tract infections significantly contribute to pediatric emergency visits and hospital admissions. Among these, bronchiolitis is the most common and clinically significant condition during infancy [1,2].

Bronchiolitis was once regarded exclusively as a viral disease; however, it is now increasingly acknowledged that environmental factors and host vulnerability critically influence its severity. Particular attention has been drawn to the role of air pollution in recent years: the heightened vulnerability of infants renders them especially susceptible to the adverse effects of airborne pollutants [3,4], such as fine particulate matter (PM2.5 and PM10), nitrogen dioxide (NO_2_), ozone (O_3_), and volatile organic compounds (VOCs) that penetrate deeply into the airways, where they can impair mucosal defenses and promote inflammation, thereby rendering young children more susceptible to respiratory infections [5]. These effects are particularly alarming during the winter season, as exposure to elevated pollution levels often coincides with the peak circulation of viruses [6,7,8]. As traditional viral patterns shift under the combined influence of warming temperatures and environmental degradation, bronchiolitis emerges as a key indicator of how climate and pollution intersect with child health.

This work explores and examines the relationship between air pollution and bronchiolitis by integrating biological mechanisms, epidemiological data, and current public health perspectives.

This narrative review does not follow a systematic search protocol; studies were selected based on their relevance to the topic, including both the recent and foundational literature.

## 2. Pathogenesis, Clinical Features, and Risk Factors

Bronchiolitis is primarily caused by Respiratory Syncytial Virus (RSV). It typically affects infants under 12 months and is a leading cause of hospital admission during the first year of life. Although typically self-limiting, the disease can progress to severe forms requiring oxygen therapy or ventilatory support, especially in preterm infants or those with underlying medical conditions [2,9]. Globally, RSV is responsible for more than 30 million cases of lower-respiratory-tract infection per year in children under five, with over 3 million hospitalizations and around 100,000 deaths, most of which occur in infants under six months of age [10].

Numerous studies have established a correlation between severe infantile bronchiolitis and an increased risk of chronic respiratory conditions, including childhood asthma. Consequently, this infection represents not only a concern in early childhood but also a condition with potential long-term health implications [11,12,13].

The infection initiates in the upper respiratory tract and subsequently disseminates to the bronchioles, where inflammation and the accumulation of mucus obstruct the airways, diminishing lung function [1,14]. RSV is recognized by airway epithelial cells through pattern recognition receptors, including TLR2 and TLR4, which leads to the activation of the MAPK and NF-κB pathways [15,16,17]. These signaling cascades result in the release of proinflammatory cytokines and chemokines, including IL-1β, IL-6, and IL-8, which promote neutrophilic infiltration and epithelial damage in the lower airways. In murine models, reactive oxygen species (ROS), generated through mitochondrial and NOX2 pathways, have been implicated in inflammasome (NLRP3) activation and IL-1β maturation, further amplifying airway inflammation [18]. Adaptive immune responses during RSV infection show a tendency toward Th2 and Th17 polarization, with elevated IL-4 and IL-17 levels contributing to mucus hypersecretion and airway hyperreactivity [19,20].

Clinical presentation typically includes tachypnea, wheezing, nasal flaring, chest retractions, and feeding difficulties. In the most vulnerable infants—especially preterms—apnea may be an early and isolated sign. Severity tends to peak between the second and fourth day of illness and may require hospitalization for supportive care. Consensus guidelines define the disease as predominantly clinical, with diagnosis based on history and physical examination, with no routine need for radiological or laboratory investigations [21]. An overview of clinical presentation and risk stratification is summarized in Table 1.

Multiple risk factors can indeed contribute to the severity of the disease. These include gestational age below 35 weeks, postnatal age under three months, underlying cardiopulmonary conditions, immunodeficiency, and the absence of breastfeeding [9]. These host-related vulnerabilities interact with external factors, including environmental conditions and exposures. Recent studies indicate that ambient air pollution—specifically fine particulate matter and nitrogen dioxide—has emerged as a significant modifier of disease expression. In fact, a systematic review conducted by Shi et al. identified several key risk factors for severe lower respiratory infections related to RSV, which include well-known factors such as young age, prematurity, low birth weight, and crowded living conditions, in addition to exposure to household air pollution [22].

There is solid evidence that indoor air pollution represents a major environmental determinant of respiratory morbidity in early childhood: exposure to combustion products from biomass or kerosene used for cooking or heating, particularly in poorly ventilated dwellings, has been associated with an increased risk of acute lower respiratory infections among children under five, especially in low-resource settings [23]; likewise, household exposure to secondhand tobacco smoke has been consistently linked to bronchiolitis, with odds ratios exceeding 2.0 in affected infants [24].

These studies support the notion that air pollution not only increases the incidence of bronchiolitis but may also worsen its clinical trajectory. Environmental sensitivity in early infancy—especially during peak viral circulation—therefore represents a critical point of intersection between host biology and urban exposure.

Coinfections between RSV and other respiratory viruses, particularly human metapneumovirus (hMPV) and rhinovirus, have been frequently reported in infants and may influence disease severity. RSV–hMPV coinfection has been associated with worse clinical outcomes, including an increased risk of intensive care unit admission, as shown in a recent meta-analysis [25]. In contrast, the same meta-analysis did not find a significant overall association between RSV–rhinovirus coinfection and disease severity, reflecting inconsistency across individual studies. However, a large observational study [26] did report that RSV–rhinovirus coinfection was independently associated with more severe clinical presentations, particularly in the presence of comorbidities. These findings highlight the need for further research to clarify the impact of specific viral coinfection patterns in both high-risk and non-high-risk populations.

## 3. Epidemiology and Seasonality of Bronchiolitis

In temperate regions, bronchiolitis typically follows a clear seasonal pattern, peaking in the winter months. Recent shifts, notably during the COVID-19 pandemic, have disrupted this seasonal pattern, leading to unexpected surges outside the usual peak periods. Non-pharmaceutical interventions, such as mask-wearing, lockdowns, and decreased social interactions, resulted in a decline in the circulation of RSV [27]. After the lifting of the restrictions, a late resurgence of bronchiolitis was observed—occurring outside the traditional epidemic months and with unexpectedly high case numbers, even during summer, anticipating the traditional season [28,29].

This phenomenon is referred to as “immunity debt”. It reflects a temporary interruption in viral exposure during a critical immunological window, which may later result in larger cohorts of susceptible infants and more pronounced outbreaks once viral transmission resumes [30,31]. In Italy, these dynamics were observed and documented in the 2021–2022 season, when a critical rebound in RSV activity led to more intense peaks in pediatric emergency visits and hospital admissions [28].

These shifts have challenged prediction models. Furthermore, climate variability can contribute to changes in viral transmission patterns; however, more research is needed to quantify these effects.

## 4. Environmental Determinants of Bronchiolitis

### 4.1. Overview of Pollutants Relevant to Respiratory Health

This section introduces the main pollutant classes relevant to pediatric respiratory health, forming the basis for the biological and epidemiological insights discussed in Section 4.2 and Section 4.3. Among the most relevant pollutants implicated in early-life respiratory vulnerability are particulate matter PM2.5 and PM10, NO_2_, O_3_, and VOCs [4,32].

Particulate matter is composed of a complex mix of solid and liquid substances suspended in air. It originates from both natural and anthropogenic sources, including vehicular traffic, industrial combustion, and biomass burning. PM2.5 particles, due to their small diameter, can reach the alveolar space and cross into systemic circulation, often carrying adsorbed toxic components such as heavy metals and polycyclic aromatic hydrocarbons [32,33].

Gaseous pollutants such as NO_2_—primarily produced by combustion engines—are known to damage epithelial cells, reduce ciliary motility, and increase airway reactivity [34]. Ozone, a secondary pollutant formed through photochemical reactions, acts primarily as an oxidative stressor, triggering inflammation and epithelial injury [32,35].

Volatile organic compounds (VOCs), such as benzene and toluene, although less studied than other pollutants, have been shown to impair epithelial barrier function [32,36,37,38,39]. These mechanisms may create a substrate where even moderate viral insults can produce a disproportionately severe clinical picture. Understanding the nature, sources, and biological effects of these pollutants is thus crucial for interpreting their impact on bronchiolitis in real-world settings. See Table 2 for a summary of the main pollutants, their sources, and associated respiratory effects.

### 4.2. Biological Mechanisms of Pollutant–Virus Interaction

The interaction between air pollutants and respiratory viruses in early infancy appears to be more than additive. Diverse experimental evidence supports the hypothesis of a synergistic relationship between pollution and viral infections.

In murine models, pre-exposure to diesel exhaust particles prior to RSV inoculation has been shown to enhance viral replication and exacerbate lung inflammation compared to the virus alone [40]. These findings highlight how pollutant-induced epithelial dysfunction creates a more permissive viral invasion and propagation environment.

Moreover, pollutants such as PM2.5 and NO_2_ alter the integrity of tight junctions between airway epithelial cells, increasing permeability and potentially facilitating viral access to the lower airways [41]. At the same time, exposure to reactive pollutants leads to oxidative stress, with overproduction of reactive oxygen species (ROS) that amplify epithelial injury and activate pro-inflammatory transcription factors [33].

Beyond direct cytotoxicity, pollution affects innate and adaptive immunity. Early-life exposure to urban air contaminants has been associated with a shift toward Th2-skewed immunity, which may impair viral clearance and favor sustained inflammation [42], alongside reduced interferon responses [43]. These alterations are particularly concerning in infants, whose immune systems are naturally biased toward tolerance and are still undergoing maturation.

Recent studies suggest that these exposures can also lead to epigenetic changes, including DNA methylation shifts and altered expression of genes involved in inflammation and repair, even in utero [44]. Additionally, prenatal exposure to air pollution has been associated with impaired alveolar development, reduced lung function, and heightened susceptibility to viral infections in animal models [45,46]. Moreover, recent cohort data show that prenatal exposure to PM2.5 has a negative impact on childhood lung function trajectories, suggesting a lasting effect beyond infancy [47].

In early infancy, several anatomical and physiological features—narrow airways, higher respiratory rate per kilogram, and immature detoxification systems—amplify the vulnerability to environmental pollutants [48,49,50]. Moreover, the prenatal and early postnatal periods represent critical windows for lung development, during which exposure to pollutants may alter alveolarization, immune maturation, and respiratory microbiota [51,52].

### 4.3. Epidemiological Evidence on Pollution and Bronchiolitis

While these insights establish biological plausibility, large-scale epidemiological studies provide the necessary population-level validation of these mechanisms. The majority of epidemiological studies on this theme rely on time-series or case-crossover designs, correlating short-term pollutant exposure with spikes in emergency visits or hospitalizations for bronchiolitis, particularly in infants under one year of age.

In Chile, Terrazas et al. documented a clear temporal association between increases in PM10 and bronchiolitis-related hospitalizations, especially during periods of high solar radiation and atmospheric stagnation [53]. In a population-based study conducted in Israel, Yitshak-Sade et al. found that infants exposed to elevated weekly concentrations of PM2.5 during early life had a significantly higher risk of hospitalization for bronchiolitis, particularly those living in high-exposure settings [54].

In France, a study by Ségala et al. established a positive association between pollutants levels and consultations and hospitalizations for bronchiolitis in infants during the winter season [55]. Similar findings have been observed in urban North America. Karr et al. demonstrated that infants residing in areas with high levels of traffic-related air pollution were more likely to be hospitalized for bronchiolitis [56]. In Massachusetts, Girguis et al. reported that acute but not chronic exposure to PM2.5 were associated with increased bronchiolitis incidence, with stronger effects observed during colder months [57,58].

Within this broader context, the Italian setting—particularly the Po Valley—illustrates how region-specific pollution patterns can amplify respiratory vulnerability in early life, similarly to what has been observed in other high-exposure areas worldwide. This area is known for persistent atmospheric stagnation and some of the highest levels of PM2.5 and NO_2_ in Europe. Several studies have explored the impact of air pollution on bronchiolitis in this context. In a large pediatric cohort in Padova, Gallo et al. observed a significant association between emergency department visits for bronchiolitis and short-term increases in PM2,5, PM10, and NO_2_ levels in infants under one year of age [59]. Carugno et al. conducted a time-series analysis on over 2800 hospitalizations for RSV bronchiolitis in infants under one year of age, demonstrating a consistent association between daily PM10 concentrations and hospitalization risk [7]. Zama et al. observed significant associations between bronchiolitis referrals and a range of air quality indicators, especially organic and elemental carbon [60]. In a 10-year study, RSV bronchiolitis hospitalizations were positively associated with air pollution levels—especially benzene, NO_2_, and PM_10_—even after adjusting for viral seasonality using monthly trends [8].

### 4.4. Dose–Response Relationships Between Pollutants and Respiratory Infections

Beyond demonstrating associations, several studies have attempted to quantify the dose–response relationship between specific pollutants and bronchiolitis risk. Short- and medium-term exposures—particularly in the two weeks preceding hospital admission for bronchiolitis—were associated with a 6–7% increased risk per 10 µg/m^3^ PM10, with the highest effect observed between lag days 0 and 11 (IRR up to 1.15, 95%CI 1.08–1.23) [7]. PM2.5 has shown comparable effects: one study found that a 10 µg/m^3^ increase in PM2.5 during the first year of life was associated with an increase in bronchiolitis hospitalizations, while a similar increase in NO_2_ was linked to a higher risk [59]. Other investigations using linear and non-linear models [61] have described strong associations during the first year of life, suggesting heightened susceptibility in early infancy. A recent systematic review by Pepper et al. [62] adds interpretative depth: while the review supports the plausibility of a dose–response association for PM2.5 during prenatal exposure, it also highlights significant heterogeneity and potential confounding, suggesting caution in generalizing findings. These considerations are complemented by a recent meta-analysis [63], which reported increased risks for all three pollutants, although statistical significance was reached only for short-term PM10 exposure.

## 5. Climate Change and the Future of Bronchiolitis

Beyond air pollutants, broader environmental shifts, such as climate change, are increasingly influencing the temporal and spatial patterns of respiratory diseases in early childhood. As global temperatures rise, the seasonality of many respiratory infections has become less predictable.

Warmer temperatures and altered rainfall patterns will contribute to the extended circulation of RSV. Prolonged warmer winters, fluctuating humidity, and increased airborne particulate matter create unpredictable viral peaks. This introduces new challenges for public health preparedness, as the conventional forecasting models, which depend on stable seasonal patterns, have been disrupted. This disruption is particularly noteworthy due to the phenomenon of immunity debt, resulting from the interrupted viral exposure during the pandemic, which has culminated in a larger population of susceptible individuals, as previously noted [31].

Additionally, extreme weather events, including heatwaves, wildfires, and floods, are becoming more frequent due to climate instability. These events exacerbate the respiratory vulnerability of infants, especially in areas with already high pollution levels. Wildfires, in particular, release large quantities of PM2.5 and other irritant gases, which contribute to respiratory inflammation and compromise mucosal defenses in infants [64,65]. In a similar manner, floods and extreme rainfall can lead to the displacement of populations into overcrowded shelters, resulting in an elevated risk of infection transmission and inadequate access to healthcare services [66]. Heat events are associated with increased respiratory morbidity, particularly among infants and young children [67].

Importantly, climate change also degrades air quality through specific feedback mechanisms. Rising temperatures intensify photochemical reactions that generate ground-level ozone, particularly during heatwaves. A recent epidemiological study in London found that ozone mediates a significant portion—up to 9.6%—of the respiratory mortality risk associated with heat events in vulnerable populations, including infants [68]. Climate-driven atmospheric stagnation also facilitates the surface accumulation of both ozone and fine particulate matter [69]. As discussed earlier, these pollutants impair the respiratory epithelium and may potentiate viral infections, contributing to more severe clinical presentations of bronchiolitis. These effects are further compounded in urban areas by emissions from traffic and industry.

A global modeling study by Baker et al. [70] demonstrated that temperature, absolute humidity, and precipitation significantly influence RSV seasonality, with climate change potentially shifting transmission peaks and altering the timing of epidemics across geographic regions. These dynamics may have implications for the optimal timing of immunoprophylaxis campaigns. Furthermore, as reviewed by Domingo et al. [71], climate stressors and air pollution are increasingly recognized as synergistic drivers of viral respiratory disease, including RSV.

Looking ahead, climate change will continue to challenge current public health frameworks. In this panorama, bronchiolitis serves as a sentinel condition, reflecting the intersection of environmental and viral factors in the broader context of a changing climate.

## 6. Strategies for Prevention and Policy Implications

Preventive strategies are currently the most promising approach to reducing RSV-related hospitalizations in infants, especially during the first months of life. From a clinical perspective, immunoprophylaxis for RSV has undergone significant evolution. For over two decades, palivizumab, a monoclonal antibody targeting the RSV fusion protein, has been used in high-risk infants such as those born prematurely or with high-risk conditions such as congenital heart disease or lung disease [72]. However, its monthly dosing schedule and high cost have limited its broader applicability in population-level prevention.

Recently, nirsevimab has emerged as a long-acting alternative, engineered to provide protection for an entire RSV season with a single intramuscular dose. Clinical trials and early real-world studies suggest that nirsevimab offers comparable or superior efficacy to palivizumab, with extended protection and simplified logistics [73,74]. Unlike palivizumab, which is restricted to high-risk groups, nirsevimab is designed for routine use in all infants, including healthy term newborns, representing a shift toward universal passive immunization against RSV. This advancement holds promise not only for reducing hospitalizations but also for alleviating pressure on the healthcare system during seasonal epidemics.

At the individual level, reducing indoor pollutant exposure is critical. Interventions such as the use of High-Efficiency Particulate Air (HEPA) filters, improved ventilation, and avoiding secondhand smoke can significantly decrease indoor air pollution, reducing respiratory symptoms in at-risk infants.

From a public health perspective, monitoring air quality and issuing real-time alerts during high-pollution days can allow for more effective protective measures. Local and national air quality networks should be integrated with healthcare data systems to anticipate hospital surges during pollution peaks, as demonstrated by several epidemiological studies linking pollution levels to bronchiolitis hospitalizations [7,59,61]. Families should be advised to limit outdoor activities during days with high air pollution, particularly in regions where PM2.5 and NO_2_ levels are often elevated.

These strategies are explicitly recommended by the World Health Organization, which has called for the adoption of early-warning tools, air quality indices, and public-facing communication systems as essential elements of respiratory disease prevention in children. Aligning with these recommendations, the WHO emphasize that multisectoral policies—spanning transport, energy, urban planning, and health—are essential to protect children’s health from environmental risks [75].

At the policy level, reducing vehicular and industrial emissions remains essential, particularly in densely populated urban areas where air quality is poorest. Implementing stricter emission standards can significantly lower concentrations of PM2.5 and O_3_ [76]. Incorporating urban green spaces not only improves air quality but also mitigates the urban heat island effect, further reducing pollution levels [77]. Recent assessments by the European Environment Agency and the Lancet Countdown confirm that air pollution remains one of the leading environmental risks to human health across Europe, exacerbated by climate-related stressors such as heatwaves and wildfires [78,79].

As viral circulation becomes increasingly erratic due to climate instability, traditional seasonal models are no longer sufficient. To anticipate and mitigate pediatric respiratory surges, we need integrated surveillance systems capable of combining viral activity, air quality data, climate trends, and healthcare utilization. These tools will support early warning mechanisms and better allocation of clinical resources. At the same time, urgent policy action is needed to reduce emissions and strengthen climate resilience, especially in urban areas where the convergence of pollution and viral exposure disproportionately affects young children. Facing these intersecting threats requires a dual approach: medical innovation to protect the most vulnerable, and structural reforms to address the environmental determinants of health.

## 7. Limitations

This narrative review is not based on a systematic search protocol, and article selection was driven by relevance rather than predefined inclusion criteria. While this approach allows for flexibility and breadth, it may introduce selection bias and limit reproducibility.

Moreover, although many studies support a dose–response relationship between air pollutants and bronchiolitis, findings are affected by heterogeneity in study design, exposure assessment, and outcome definitions. Most available evidence is observational and subject to residual confounding, especially from socioeconomic factors, indoor exposures, and care practices.

In addition, real-world exposures are multifactorial, and the co-occurrence of pollutants, infections, and climatic stressors complicates attribution of risk to specific agents. The lack of longitudinal, individual-level data with mechanistic outcomes also limits causal interpretation and understanding of long-term effects.

Lastly, generalizability is constrained by regional differences in pollution profiles, healthcare systems, and viral circulation patterns, particularly in underrepresented low- and middle-income settings.

## 8. Conclusions and Future Directions

The cumulative evidence presented in this review underscores the necessity to reconceptualize bronchiolitis as a condition shaped not only by viral epidemiology but also by environmental determinants that modulate host susceptibility, disease trajectory, and long-term complications. The interplay between ambient pollutants and climate-induced shifts in viral circulation contributes to the observed variability in the incidence, severity, and timing of bronchiolitis outbreaks. These trends are becoming increasingly difficult to predict using classical seasonal models. While targeted immunoprophylaxis, such as nirsevimab, offers a significant advancement in RSV-specific prevention, it does not address the full spectrum of risk factors, particularly those related to environmental exposure. This gap highlights the importance of integrating pediatric respiratory health into broader air quality and climate adaptation frameworks. Future research should prioritize standardized exposure assessment methods and longitudinal cohort designs to clarify dose–response relationships and disentangle the complex effects of co-exposures such as tobacco smoke and urban pollution. Moreover, ethical and equity dimensions must not be overlooked; children from socioeconomically disadvantaged backgrounds often face disproportionate exposure to poor air quality and have limited access to protective interventions. In an era of increasing ecological and atmospheric instability, bronchiolitis serves as a reminder that the respiratory health of the youngest reflects the air they breathe and the choices we make as a society to protect it.

## Figures and Tables

**Table 1:** Clinical presentation and risk stratification in bronchiolitis.

Category	Details
Typical age	Infants <12 months, peak incidence <6 months.
Common agents	RSV (most common), rhinovirus, hMPV, and other respiratory viruses.
Clinical presentation	Nasal congestion, tachypnea, wheezing, chest retractions, and feeding difficulty. In young or preterm infants, apnea may be the first sign.
Markers of severity	Hypoxia (SpO_2_ < 90–92%), signs of respiratory distress, apnea, feeding refusal, and cyanosis.
Risk factors for severe course	Prematurity, age <3 months, chronic lung disease, congenital heart disease, immunodeficiency, no breastfeeding, and exposure to tobacco smoke or pollutants.
Recommended management	Supportive care: oxygen, hydration, and nutritional support if needed. No routine use of bronchodilators, steroids, or antibiotics.

**Table 2:** Key pollutants implicated in bronchiolitis: sources, effects, and evidence.

Pollutant	Primary Sources	Respiratory Effects	Evidence in Bronchiolitis
PM2.5/PM10	Traffic, industry, and biomass combustion	Penetration into lower airways, oxidative stress, and inflammation	Increased hospital visits
NO_2_	Combustion engines (diesel and domestic heating)	Epithelial injury, reduced ciliary clearance, and pro-inflammatory effect	Link with RSV bronchiolitis severity
O_3_	Photochemical reactions involving NOx + VOCs	Oxidative damage and airway hyperresponsiveness	Less directly studied, but plausible synergistic effects
VOCs	Fuels, solvents, and industrial emissions	Mucosal irritation	Under-investigated in bronchiolitis; potentially relevant indoors and outdoors

## Data Availability

The data are contained within the article.

## Abbreviations

The following abbreviations are used in this manuscript:

RSV	Respiratory Syncytial Virus
PM	Particulate Matter
VOC	Volatile Organic Compound

## References

[1] Florin T.A., Plint A.C., Zorc J.J. (2017). Viral Bronchiolitis. Lancet.

[2] Dalziel S.R., Haskell L., O’Brien S., Borland M.L., Plint A.C., Babl F.E., Oakley E. (2022). Bronchiolitis. Lancet.

[3] Landrigan P.J., Fuller R., Fisher S., Suk W.A., Sly P., Chiles T.C., Bose-O’Reilly S. (2019). Pollution and Children’s Health. Sci. Total Environ..

[4] Salvi S. (2007). Health Effects of Ambient Air Pollution in Children. Paediatr. Respir. Rev..

[5] Esposito S., Fainardi V., Titolo A., Lazzara A., Menzella M., Campana B., Argentiero A., Principi N. (2025). How Air Pollution Fuels Respiratory Infections in Children: Current Insights. Front. Public Health.

[6] Karr C., Lumley T., Shepherd K., Davis R., Larson T., Ritz B., Kaufman J. (2006). A Case–Crossover Study of Wintertime Ambient Air Pollution and Infant Bronchiolitis. Environ. Health Perspect..

[7] Carugno M., Dentali F., Mathieu G., Fontanella A., Mariani J., Bordini L., Milani G.P., Consonni D., Bonzini M., Bollati V. (2018). PM10 Exposure Is Associated with Increased Hospitalizations for Respiratory Syncytial Virus Bronchiolitis among Infants in Lombardy, Italy. Environ. Res..

[8] Nenna R., Evangelisti M., Frassanito A., Scagnolari C., Pierangeli A., Antonelli G., Nicolai A., Arima S., Moretti C., Papoff P. (2017). Respiratory Syncytial Virus Bronchiolitis, Weather Conditions and Air Pollution in an Italian Urban Area: An Observational Study. Environ. Res..

[9] Baraldi E., Lanari M., Manzoni P., Rossi G.A., Vandini S., Rimini A., Romagnoli C., Colonna P., Biondi A., Biban P. (2014). Inter-Society Consensus Document on Treatment and Prevention of Bronchiolitis in Newborns and Infants. Ital. J. Pediatr..

[10] Shi T., McAllister D.A., O’Brien K.L., Simoes E.A.F., Madhi S.A., Gessner B.D., Polack F.P., Balsells E., Acacio S., Aguayo C. (2017). Global, Regional, and National Disease Burden Estimates of Acute Lower Respiratory Infections Due to Respiratory Syncytial Virus in Young Children in 2015: A Systematic Review and Modelling Study. Lancet.

[11] Fauroux B., Simões E.A.F., Checchia P.A., Paes B., Figueras-Aloy J., Manzoni P., Bont L., Carbonell-Estrany X. (2017). The Burden and Long-Term Respiratory Morbidity Associated with Respiratory Syncytial Virus Infection in Early Childhood. Infect. Dis. Ther..

[12] Coutts J., Fullarton J., Morris C., Grubb E., Buchan S., Rodgers-Gray B., Thwaites R. (2020). Association between Respiratory Syncytial Virus Hospitalization in Infancy and Childhood Asthma. Pediatr. Pulmonol..

[13] Sigurs N., Aljassim F., Kjellman B., Robinson P.D., Sigurbergsson F., Bjarnason R., Gustafsson P.M. (2010). Asthma and Allergy Patterns over 18 Years after Severe RSV Bronchiolitis in the First Year of Life. Thorax.

[14] Meissner H.C. (2016). Viral Bronchiolitis in Children. N. Engl. J. Med..

[15] Segovia J., Sabbah A., Mgbemena V., Tsai S.-Y., Chang T.-H., Berton M.T., Morris I.R., Allen I.C., Ting J.P.-Y., Bose S. (2012). TLR2/MyD88/NF-κB Pathway, Reactive Oxygen Species, Potassium Efflux Activates NLRP3/ASC Inflammasome during Respiratory Syncytial Virus Infection. PLoS ONE.

[16] Haynes L.M., Moore D.D., Kurt-Jones E.A., Finberg R.W., Anderson L.J., Tripp R.A. (2001). Involvement of Toll-Like Receptor 4 in Innate Immunity to Respiratory Syncytial Virus. J. Virol..

[17] Kurt-Jones E.A., Popova L., Kwinn L., Haynes L.M., Jones L.P., Tripp R.A., Walsh E.E., Freeman M.W., Golenbock D.T., Anderson L.J. (2000). Pattern Recognition Receptors TLR4 and CD14 Mediate Response to Respiratory Syncytial Virus. Nat. Immunol..

[18] Malinczak C.-A., Schuler C.F., Duran A.J., Rasky A.J., Mire M.M., Núñez G., Lukacs N.W., Fonseca W. (2021). NLRP3-Inflammasome Inhibition during Respiratory Virus Infection Abrogates Lung Immunopathology and Long-Term Airway Disease Development. Viruses.

[19] Lotz M.T., Peebles R.S. (2012). Mechanisms of Respiratory Syncytial Virus Modulation of Airway Immune Responses. Curr. Allergy Asthma Rep..

[20] Mukherjee S., Lindell D.M., Berlin A.A., Morris S.B., Shanley T.P., Hershenson M.B., Lukacs N.W. (2011). IL-17–Induced Pulmonary Pathogenesis during Respiratory Viral Infection and Exacerbation of Allergic Disease. Am. J. Pathol..

[21] Ralston S.L., Lieberthal A.S., Meissner H.C., Alverson B.K., Baley J.E., Gadomski A.M., Johnson D.W., Light M.J., Maraqa N.F., Mendonca E.A. (2014). Clinical Practice Guideline: The Diagnosis, Management, and Prevention of Bronchiolitis. Pediatrics.

[22] Shi T., Balsells E., Wastnedge E., Singleton R., Rasmussen Z.A., Zar H.J., Rath B.A., Madhi S.A., Campbell S., Vaccari L.C. (2015). Risk Factors for Respiratory Syncytial Virus Associated with Acute Lower Respiratory Infection in Children under Five Years: Systematic Review and Meta–Analysis. J. Glob. Health.

[23] Sharma S., Sethi G.R., Rohtagi A., Chaudhary A., Shankar R., Joshi V., Sapir D.G. (1998). Indoor Air Quality and Acute Lower Respiratory Infection in Indian Urban Slums. Environ. Health Perspect..

[24] Jones L.L., Hashim A., McKeever T., Cook D.G., Britton J., Leonardi-Bee J. (2011). Parental and Household Smoking and the Increased Risk of Bronchitis, Bronchiolitis and Other Lower Respiratory Infections in Infancy: Systematic Review and Meta-Analysis. Respir. Res..

[25] Li Y., Pillai P., Miyake F., Nair H. (2020). The Role of Viral Co-Infections in the Severity of Acute Respiratory Infections among Children Infected with Respiratory Syncytial Virus (RSV): A Systematic Review and Meta-Analysis. J. Glob. Health.

[26] Comte A., Bour J.-B., Darniot M., Pitoiset C., Aho-Glélé L.S., Manoha C. (2020). Epidemiological Characteristics and Clinical Outcomes of Human Rhinovirus Infections in a Hospitalized Population. Severity Is Independently Linked to RSV Coinfection and Comorbidities. J. Clin. Virol..

[27] Baker R.E., Park S.W., Yang W., Vecchi G.A., Metcalf C.J.E., Grenfell B.T. (2020). The Impact of COVID-19 Nonpharmaceutical Interventions on the Future Dynamics of Endemic Infections. Proc. Natl. Acad. Sci. USA.

[28] Ghirardo S., Ullmann N., Zago A., Ghezzi M., Minute M., Madini B., D’Auria E., Basile C., Castelletti F., Chironi F. (2024). Increased Bronchiolitis Burden and Severity after the Pandemic: A National Multicentric Study. Ital. J. Pediatr..

[29] Brisca G., Mariani M., Buratti S., Ferretti M., Pirlo D., Buffoni I., Mallamaci M., Salvati P., Tagliarini G., Piccotti E. (2023). How Has the SARS-CoV-2 Pandemic Changed the Epidemiology and Management of Acute Bronchiolitis?. Pediatr. Pulmonol..

[30] Lenglart L., Titomanlio L., Bognar Z., Bressan S., Buonsenso D., De T., Farrugia R., Honeyford K., Maconochie I.K., Moll H.A. (2024). Surge of Pediatric Respiratory Tract Infections After the COVID-19 Pandemic and the Concept of “Immune Debt”. J. Pediatr..

[31] Cohen R., Ashman M., Taha M.-K., Varon E., Angoulvant F., Levy C., Rybak A., Ouldali N., Guiso N., Grimprel E. (2021). Pediatric Infectious Disease Group (GPIP) Position Paper on the Immune Debt of the COVID-19 Pandemic in Childhood, How Can We Fill the Immunity Gap?. Infect. Dis. Now..

[32] Manisalidis I., Stavropoulou E., Stavropoulos A., Bezirtzoglou E. (2020). Environmental and Health Impacts of Air Pollution: A Review. Front. Public Health.

[33] Anderson J.O., Thundiyil J.G., Stolbach A. (2012). Clearing the Air: A Review of the Effects of Particulate Matter Air Pollution on Human Health. J. Med. Toxicol..

[34] Chen T.-M., Kuschner W.G., Gokhale J., Shofer S. (2007). Outdoor Air Pollution: Nitrogen Dioxide, Sulfur Dioxide, and Carbon Monoxide Health Effects. Am. J. Med. Sci..

[35] Wu T., Li Z., Wei Y. (2023). Advances in Understanding Mechanisms Underlying Mitochondrial Structure and Function Damage by Ozone. Sci. Total Environ..

[36] Wang B., Yu L., Liu W., Yang M., Fan L., Zhou M., Ma J., Wang X., Nie X., Cheng M. (2022). Cross-Sectional and Longitudinal Associations of Acrolein Exposure with Pulmonary Function Alteration: Assessing the Potential Roles of Oxidative DNA Damage, Inflammation, and Pulmonary Epithelium Injury in a General Adult Population. Environ. Int..

[37] Hu Y., Niu Z., Cao C., Gao J., Pan M., Cai Y., Zhao Z. (2024). Volatile Organic Compounds (VOC) Metabolites in Urine Are Associated with Increased Systemic Inflammation Levels, and Smokers Are Identified as a Vulnerable Population. Ecotoxicol. Environ. Saf..

[38] Conklin D.J. (2016). Acute Cardiopulmonary Toxicity of Inhaled Aldehydes: Role of TRPA1. Ann. N. Y. Acad. Sci..

[39] Henning R.J., Johnson G.T., Coyle J.P., Harbison R.D. (2017). Acrolein Can Cause Cardiovascular Disease: A Review. Cardiovasc. Toxicol..

[40] Harrod K.S., Jaramillo R.J., Rosenberger C.L., Wang S.-Z., Berger J.A., McDonald J.D., Reed M.D. (2003). Increased Susceptibility to RSV Infection by Exposure to Inhaled Diesel Engine Emissions. Am. J. Respir. Cell Mol. Biol..

[41] Sözener Z.C. (2020). Environmental Factors in Epithelial Barrier Dysfunction. J. Allergy Clin. Immunol..

[42] Salvi S. (2001). Pollution and Allergic Airways Disease. Curr. Opin. Allergy Clin. Immunol..

[43] Allouche J., Cremoni M., Brglez V., Graça D., Benzaken S., Zorzi K., Fernandez C., Esnault V., Levraut M., Oppo S. (2022). Air Pollution Exposure Induces a Decrease in Type II Interferon Response: A Paired Cohort Study. eBioMedicine.

[44] Isaevska E., Moccia C., Asta F., Cibella F., Gagliardi L., Ronfani L., Rusconi F., Stazi M.A., Richiardi L. (2021). Exposure to Ambient Air Pollution in the First 1000 Days of Life and Alterations in the DNA Methylome and Telomere Length in Children: A Systematic Review. Environ. Res..

[45] Veras M.M., De Oliveira Alves N., Fajersztajn L., Saldiva P. (2017). Before the First Breath: Prenatal Exposures to Air Pollution and Lung Development. Cell Tissue Res..

[46] Lau C., Behlen J.C., Myers A., Li Y., Zhao J., Harvey N., Wright G., Hoffmann A.R., Zhang R., Johnson N.M. (2022). In Utero Ultrafine Particulate Exposure Yields Sex- and Dose-Specific Responses to Neonatal Respiratory Syncytial Virus Infection. Environ. Sci. Technol..

[47] Usemann J., Mozun R., Kuehni C.E., De Hoogh K., Flueckiger B., Singer F., Zwahlen M., Moeller A., Latzin P., LUIS Study Group (2024). Air Pollution Exposure during Pregnancy and Lung Function in Childhood: The LUIS Study. Pediatr. Pulmonol..

[48] Bennett W.D., Zeman K.L., Jarabek A.M. (2008). Nasal Contribution to Breathing and Fine Particle Deposition in Children Versus Adults. J. Toxicol. Environ. Health Part A.

[49] Bateson T.F., Schwartz J. (2008). Children’s Response to Air Pollutants. J. Toxicol. Environ. Health Part A.

[50] Fleming S., Thompson M., Stevens R., Heneghan C., Plüddemann A., Maconochie I., Tarassenko L., Mant D. (2011). Normal Ranges of Heart Rate and Respiratory Rate in Children from Birth to 18 Years of Age: A Systematic Review of Observational Studies. Lancet.

[51] Ji J., Sun Q., Nie D., Wang Q., Zhang H., Qin F., Wang Q., Lu S., Pang G., Lu Z. (2021). Probiotics Protect against RSV Infection by Modulating the Microbiota-Alveolar-Macrophage Axis. Acta Pharmacol. Sin..

[52] Grigg J. (2009). Particulate Matter Exposure in Children: Relevance to Chronic Obstructive Pulmonary Disease. Proc. Am. Thorac. Soc..

[53] Terrazas C., Castro-Rodriguez J.A., Camargo C.A., Borzutzky A. (2019). Solar Radiation, Air Pollution, and Bronchiolitis Hospitalizations in Chile: An Ecological Study. Pediatr. Pulmonol..

[54] Yitshak-Sade M., Yudovitch D., Novack V., Tal A., Kloog I., Goldbart A. (2017). Air Pollution and Hospitalization for Bronchiolitis among Young Children. Ann. Am. Thorac. Soc..

[55] Karr C.J., Rudra C.B., Miller K.A., Gould T.R., Larson T., Sathyanarayana S., Koenig J.Q. (2009). Infant exposure to fine particulate matter and traffic and risk of hospitalization for RSV bronchiolitis in a region with lower ambient air pollution. Environ. Res..

[56] Ségala C., Poizeau D., Mesbah M., Willems S., Maidenberg M. (2008). Winter Air Pollution and Infant Bronchiolitis in Paris. Environ. Res..

[57] Girguis M.S., Strickland M.J., Hu X., Liu Y., Chang H.H., Kloog I., Belanoff C., Bartell S.M., Vieira V.M. (2018). Exposure to Acute Air Pollution and Risk of Bronchiolitis and Otitis Media for Preterm and Term Infants. J. Expo. Sci. Environ. Epidemiol..

[58] Girguis M.S., Strickland M.J., Hu X., Liu Y., Chang H.H., Belanoff C., Bartell S.M., Vieira V.M. (2017). Chronic PM2.5 Exposure and Risk of Infant Bronchiolitis and Otitis Media Clinical Encounters. Int. J. Hyg. Environ. Health.

[59] Gallo E., Bressan S., Baraldo S., Bottigliengo D., Geremia S., Acar A.S., Zagolin L., Marson G., Da Dalt L., Gregori D. (2023). Increased Risk of Emergency Department Presentations for Bronchiolitis in Infants Exposed to Air Pollution. Risk Anal..

[60] Zama D., Paccapelo A., Betti L., Manieri E., Paglione M., Rinaldi M., Dondi A., Battelli E., Biagi C., Marchegiani Rizzolli C. (2025). The Influence of Air Pollutants on the Risk of Emergency Department Presentations of Infants with Bronchiolitis in an European Air Quality Hotspot. Pediatr. Allergy Immunol..

[61] Milani G.P., Cafora M., Favero C., Luganini A., Carugno M., Lenzi E., Pistocchi A., Pinatel E., Pariota L., Ferrari L. (2022). PM_2.5_, PM_10_ and Bronchiolitis Severity: A Cohort Study. Pediatr. Allergy Immunol..

[62] Pepper M., Rebouças P., Falcão I.R., Sanchez Clemente N., Lowe R., Schneider R., Pescarini J.M., Santos G.F.D., Andrade R.F., Cortes T.R. (2025). Prenatal Exposure to Ambient Air Pollution and Subsequent Risk of Lower Respiratory Tract Infections in Childhood and Adolescence: A Systematic Review. Int. J. Hyg. Environ. Health.

[63] Comotti A., Alberti I., Spolidoro G.C.I., Vassilopoulou E., Agostoni C., Bonzini M., Carugno M., Milani G.P. (2025). Air Pollution and Hospitalization Risk in Infants with Bronchiolitis: A Systematic Review and Meta-analysis. Pediatr. Allergy Immunol..

[64] Henry S., Ospina M.B., Dennett L., Hicks A. (2021). Assessing the Risk of Respiratory-Related Healthcare Visits Associated with Wildfire Smoke Exposure in Children 0–18 Years Old: A Systematic Review. Int. J. Environ. Res. Public Health.

[65] Moore L.E., Oliveira A., Zhang R., Behjat L., Hicks A. (2023). Impacts of Wildfire Smoke and Air Pollution on a Pediatric Population with Asthma: A Population-Based Study. Int. J. Environ. Res. Public Health.

[66] The Lancet Respiratory Medicine (2024). Flooding and Excessive Rainfall Risk Respiratory Health. Lancet Respir. Med..

[67] Cheng J., Xu Z., Bambrick H., Prescott V., Wang N., Zhang Y., Su H., Tong S., Hu W. (2019). Cardiorespiratory Effects of Heatwaves: A Systematic Review and Meta-Analysis of Global Epidemiological Evidence. Environ. Res..

[68] Gao J., Wood D., Katsouyanni K., Benmarhnia T., Evangelopoulos D. (2025). The Synergistic and Mediating Effects of Ozone on Associations between High Temperature, Heatwaves and Mortality in the Greater London Area between 2010 and 2018. Environ. Res..

[69] Zhou M., Xie Y., Wang C., Shen L., Mauzerall D.L. (2024). Impacts of Current and Climate Induced Changes in Atmospheric Stagnation on Indian Surface PM2.5 Pollution. Nat. Commun..

[70] Baker R.E., Mahmud A.S., Wagner C.E., Yang W., Pitzer V.E., Viboud C., Vecchi G.A., Metcalf C.J.E., Grenfell B.T. (2019). Epidemic Dynamics of Respiratory Syncytial Virus in Current and Future Climates. Nat. Commun..

[71] Domingo K.N., Gabaldon K.L., Hussari M.N., Yap J.M., Valmadrid L.C., Robinson K., Leibel S. (2024). Impact of Climate Change on Paediatric Respiratory Health: Pollutants and Aeroallergens. Eur. Respir. Rev..

[72] Garegnani L., Styrmisdóttir L., Roson Rodriguez P., Escobar Liquitay C.M., Esteban I., Franco J.V. (2021). Palivizumab for Preventing Severe Respiratory Syncytial Virus (RSV) Infection in Children. Cochrane Database Syst. Rev..

[73] Hammitt L.L., Dagan R., Yuan Y., Baca Cots M., Bosheva M., Madhi S.A., Muller W.J., Zar H.J., Brooks D., Grenham A. (2022). Nirsevimab for Prevention of RSV in Healthy Late-Preterm and Term Infants. N. Engl. J. Med..

[74] Sumsuzzman D.M., Wang Z., Langley J.M., Moghadas S.M. (2025). Real-World Effectiveness of Nirsevimab against Respiratory Syncytial Virus Disease in Infants: A Systematic Review and Meta-Analysis. Lancet Child Adolesc. Health.

[75] World Health Organization (2019). Prescribing Clean Air: Addressing Air Pollution to Improve Child Health.

[76] Yin L., Bai B., Zhang B., Zhu Q., Di Q., Requia W.J., Schwartz J.D., Shi L., Liu P. (2025). Regional-Specific Trends of PM2.5 and O3 Temperature Sensitivity in the United States. npj Clim. Atmos. Sci..

[77] World Health Organization (2021). Air Quality Guidelines: Global Update 2021: Particulate Matter (PM2.5 and PM10), Ozone, Nitrogen Dioxide, Sulfur Dioxide and Carbon Monoxide.

[78] Romanello M., Walawender M., Hsu S.-C., Moskeland A., Palmeiro-Silva Y., Scamman D., Ali Z., Ameli N., Angelova D., Ayeb-Karlsson S. (2024). The 2024 Report of the Lancet Countdown on Health and Climate Change: Facing Record-Breaking Threats from Delayed Action. Lancet.

[79] European Environment Agency (2023). Europe’s Air Quality Status 2023—Assessment of Health Impacts from Air Pollution.

